# The association between autoimmune disease and 30-day mortality among sepsis ICU patients: a cohort study

**DOI:** 10.1186/s13054-019-2357-1

**Published:** 2019-03-18

**Authors:** Mallory Sheth, Corey M. Benedum, Leo Anthony Celi, Roger G. Mark, Natasha Markuzon

**Affiliations:** 1 MIT Operations Research Center, Cambridge, MA USA; 2 Harvard-MIT Division of Health Science and Technology, Boston, MA USA; 3 Beth Israel Deaconess Medical Center, Boston, MA USA; 4 The Charles Stark Draper Laboratory, Cambridge, MA USA; 5 Boston University School of Public Health, Boston, MA USA

**Keywords:** (3–10)-sepsis, Mortality, Autoimmune disease, Large observational database, MIMIC III, Mediation, Disease-modifying antirheumatic drugs

## Abstract

**Introduction:**

Sepsis results from a dysregulated host response to an infection that is associated with an imbalance between pro- and anti-inflammatory cytokines. This imbalance is hypothesized to be a driver of patient mortality. Certain autoimmune diseases modulate the expression of cytokines involved in the pathophysiology of sepsis. However, the outcomes of patients with autoimmune disease who develop sepsis have not been studied in detail. The objective of this study is to determine whether patients with autoimmune diseases have different sepsis outcomes than patients without these comorbidities.

**Methods:**

Using the Multiparameter Intelligent Monitoring in Intensive Care III database (v. 1.4) which contains retrospective clinical data for over 50,000 adult ICU stays, we compared 30-day mortality risk for sepsis patients with and without autoimmune disease. We used logistic regression models to control for known confounders, including demographics, disease severity, and immunomodulation medications. We used mediation analysis to evaluate how the chronic use of immunomodulation medications affects the relationship between autoimmune disease and 30-day mortality.

**Results:**

Our study found a statistically significant 27.00% reduction in the 30-day mortality risk associated with autoimmune disease presence. This association was found to be the strongest (OR 0.71, 95% CI 0.54–0.93, *P* = 0.014) among patients with septic shock. The autoimmune disease-30-day mortality association was not mediated through the chronic use of immunomodulation medications (indirect effect OR 1.07, 95% CI 1.01–1.13, *P* = 0.020).

**Conclusions:**

We demonstrated that autoimmune diseases are associated with a lower 30-day mortality risk in sepsis. Our findings suggest that autoimmune diseases affect 30-day mortality through a mechanism unrelated to the chronic use of immunomodulation medications. Since this study was conducted within a single study center, research using data from other medical centers will provide further validation.

**Electronic supplementary material:**

The online version of this article (10.1186/s13054-019-2357-1) contains supplementary material, which is available to authorized users.

## Introduction

Sepsis is a systemic inflammatory response to an infection that can lead to organ failure and death. Severe sepsis accounts for around 10.00% of all intensive care unit (ICU) admissions in the USA, and mortality rates are commonly reported between 28.00 and 50.00% [1, 2]. Sepsis outcomes and mortality have been a major focus of research over the last few years as it pertains to quality improvement and health outcomes.

Sepsis results from a dysregulated host response to an infection that is associated with an imbalance between pro- and anti-inflammatory cytokines [3]. The overactive pro-inflammatory response has been considered a primary driver of sepsis mortality; however, therapies targeting this response have not been successful in human trials [4]. Given these failures, new hypotheses have been proposed which highlight the dysregulation of both pro- and anti-inflammatory pathways [5].

Autoimmune diseases are a group of diseases that arise from an abnormal immune response of the host against substances and tissues normally present in the body. Certain autoimmune diseases have been associated with the over- or underexpression of pro- or anti-inflammatory cytokines involved in the pathophysiology of sepsis [3, 6–12]. Variations in cytokine levels may affect sepsis survival among autoimmune patients. However, there is little information regarding the physiology and outcomes of sepsis in patients with autoimmune diseases.

Examining sepsis outcomes among patients with autoimmune diseases may yield new insights into how the immune system responds to infections given different baseline cytokine levels [13]. The effect of autoimmune disease has been speculated to lead to worse clinical outcomes among patients diagnosed with sepsis as a result of the modulated immune response associated with autoimmune diseases and their treatment [14–17]. However, recent research indicates the contrary, where in certain cases patients with autoimmune diseases may have better sepsis-related clinical outcomes [13]. Yet the underlying cause of these improved clinical outcomes remains unknown.

The objective of this study is to determine the effect of autoimmune disease on sepsis outcomes. We compare 30-day mortality rates for sepsis patients with autoimmune disease and without autoimmune disease, controlling for known confounders.

## Methods

### Data source

This study used the publicly available Multiparameter Intelligent Monitoring in Intensive Care (MIMIC) III database version 1.4 [18]. MIMIC III contains de-identified clinical data for over 50,000 adult ICU stays at Beth Israel Deaconess Medical Center in Boston, MA, from 2001 to 2012 [18], and was jointly developed by the Massachusetts Institute of Technology, Phillips Healthcare, and Beth Israel Deaconess Medical Center. Any researcher who adheres to the data use requirements is permitted to use the database.

### Patient population

The primary study population consists of adult ICU patients with sepsis. We identified a *sepsis* population according to the Martin criteria, a widely used approach for identifying sepsis in administrative health data [19]. We identified a *septic shock* subpopulation—a cohort of patients meeting both sepsis and hypotension criteria indicative of substantial medical distress. We defined hypotension as three consecutive mean arterial blood pressure readings below 65 mmHg in a 30-min period or at least one dose of vasopressors during the ICU stay [20].

All patients were required to have at least 24 h of ICU data, and we selected the last ICU stay meeting these criteria for each patient. We identified 6200 patients in the database meeting the sepsis inclusion criterion and 4190 meeting the septic shock criteria.

### Predictor and outcome variables

Throughout this study, autoimmune disease refers to a set of related conditions, defined using ICD-9-CM diagnosis codes and free text analysis of the patient discharge summaries (Table 1). These conditions were selected based upon their association with the over- or underexpression of pro- or anti-inflammatory cytokines involved in the pathophysiology of sepsis [3, 6–12] (Additional file 1: Table S1). In total, we identified 496 septic patients with autoimmune disease, with rheumatoid arthritis and Crohn’s disease being the most prevalent conditions.

**Table 1:** Conditions included in autoimmune definition

Autoimmune condition	ICD-9-CM	Sepsis	Septic shock
All autoimmune conditions	–	*N* = 496	*N* = 328
Rheumatoid arthritis	714	130 (26.21%)	92 (28.05%)
Crohn’s disease	555	114 (22.98%)	85 (25.91%)
Ulcerative colitis	556.5, 556.6, 556.8, 556.9	86 (17.34%)	57 (17.38%)
Multiple sclerosis	340	64 (12.90%)	40 (12.20%)
Systemic lupus erythematosus	710.0	52 (10.48%)	40 (12.20%)
Ankylosing spondylitis	720	23 (4.64%)	16 (4.88%)
Psoriatic arthritis	696.0	20 (4.03%)	11 (3.35%)
Myasthenia gravis	358.0	16 (3.23%)	9 (2.74%)
Inflammatory myopathies	710.4, 710.3, 359.71	12 (2.41%)	6 (1.83%)
Polymyositis	710.4	9 (1.80%)	5 (1.52%)
Dermatomyositis	710.3	1 (0.20%)	1 (0.30%)
Inclusion body myositis	359.71	2 (0.40%)	0 (0.00%)
Giant cell arteritis	446.5	11 (2.22%)	4 (1.22%)
Systemic sclerosis	710.1	10 (2.01%)	7 (2.13%)
Scleroderma	701.1	9 (1.81%)	7 (2.13%)

For each patient in the study, we extracted several confounding factors from data stored in the MIMIC III database. They included age, race, sex, infection site (pulmonary or non-pulmonary), documented bacteremia, Sequential Organ Failure Assessment (SOFA) score at ICU admission, Elixhauser comorbidity index at hospital admission, ICU care unit, and pre-admission chronic conventional-synthetic and biologic disease-modifying antirheumatic drug (DMARD; Additional file 2: Table S2) and prednisone usage. SOFA includes information about the condition of a patient’s respiratory, renal, and cardiovascular systems, among others, and has been found to be a strong predictor of prognosis for ICU patients with sepsis [21]. The Elixhauser comorbidity index is a measure of disease burden that was specifically developed for use with administrative health data [22].

The primary outcome of interest in this study is patients’ 30-day mortality. Thirty-day mortality is based on data from the Social Security Death Index and reflects deaths within a 30-day window after the patient’s hospital discharge date as well as in-hospital deaths.

### Statistical analysis and modeling

In the primary analysis, we estimated relative risks with odds ratios (OR) and 95% confidence intervals (CI) for patients with autoimmune disease, compared with patients without autoimmune disease using a multivariable logistic regression model [23]. ORs were adjusted for potential confounders using two approaches: (1) all potential confounders were included in the final model and (2) only potential confounders that meaningfully affected model estimates were included in the final model. In the second approach, individual factors were added into the model one at a time, and if a variable changed the model estimate by more than 10.00%, it was retained in the final model [24, 25]. Factors that were considered as potential confounders included the following: age, race (non-Hispanic white, non-Hispanic black, Hispanic, Asian/Pacific Islander, other, missing), infection site, documented bacteremia, SOFA score, Elixhauser comorbidity index (and the individual comorbidities included in the index), ICU care unit, and chronic pre-admission DMARD or prednisone use. We also performed a survival analysis for the five most frequently reported autoimmune diseases (rheumatoid arthritis, Crohn’s disease, ulcerative colitis, multiple sclerosis, and systemic lupus erythematosus). In this analysis, we used Cox Proportional-Hazards models [24, 25] with both confounder adjustment approaches (discussed above) and report the ratio of the hazard functions, or hazards ratio (HR). To further evaluate study results, we performed additional analyses where we stratified results by the over- and underexpression of sepsis-related cytokines, assessed the joint effect of autoimmune disease and chronic DMARD or prednisone use on 30-day mortality, and performed all aforementioned analyses using the septic shock cohort.

Furthermore, for both cohorts, we performed a mediation analysis treating chronic pre-admission DMARD or prednisone usage as a potential mediator [26]. Both medications are used to treat autoimmune diseases and are strongly associated with autoimmune disease presence (DMARD OR 11.45, 95% CI 8.16–16.07; prednisone OR 4.66, 95% CI 3.67–5.90). Both medications have also been associated with an increased risk for mortality [27, 28]. The mediation analysis assumes a causal relationship between the exposure (autoimmune diseases) and mediator (chronic DMARD or prednisone usage) variables which in turn may affect the outcome (30-day mortality). Mediation analysis clarifies the nature of the relationship between the exposure and mediator to better understand the pathways by which the exposure potentially affects the outcome [26, 29]. We used the mediation analysis to estimate the direct effect of autoimmune disease on 30-day mortality and the effect of autoimmune disease on 30-day mortality that is mediated by chronic DMARD and prednisone use (indirect effect) [26].

We performed all statistical analyses using the R programming language version 3.3.3 [30] and SAS 9.4 software (SAS Institute, Inc., Cary, North Carolina) [31].

## Results

### Study population

The study cohort consisted of 6200 patients with sepsis. Of these, 496 (8.00%) had at least one autoimmune disease. To understand how the autoimmune and non-autoimmune patient populations differed from each other, we evaluated each group’s baseline characteristics (Table 2). Generally, those with an autoimmune disease were more likely to be white, younger, female; had a lower SOFA score; chronically use DMARDs or prednisone; and did not have a reported pulmonary infection.

**Table 2:** Baseline characteristics for sepsis patients, stratified by the presence of autoimmune disease

	Autoimmune disease	No autoimmune disease	*P* value
Number of patients	496	5704	
Patient outcomes			
30-day mortality	26.61%	34.55%	< 0.001
Patient characteristics			
Age (mean ± SD)	64.46 ± 14.64	66.21 ± 16.48	0.012
Sex (% male)	44.56%	57.50%	< 0.001
Race			0.027
White, non-Hispanic	80.04%	71.49%	
Black, non-Hispanic	7.26%	9.34%	
Hispanic	2.02%	3.30%	
Asian/Pacific Islander	1.61%	3.00%	
Other	2.42%	2.58%	
Unknown	6.65%	10.29%	
SOFA at admission (mean ± SD)	5.63 ± 3.67	6.50 ± 3.80	< 0.001
Elixhauser comorbidity index (mean ± SD)	9.46 ± 7.74	9.75 ± 7.83	0.411
Infection site (% pulmonary)	32.06%	39.00%	0.003
Documented bacteremia (% yes)	16.13%	18.92%	0.142
Chronic pre-admission DMARD or prednisone use	56.05%	19.78%	< 0.001
Chronic prednisone use	41.73%	17.36%	
Chronic DMARD use	34.48%	5.80%	
ICU care unit			0.694
MICU	63.91%	62.34%	
SICU	21.57%	21.27%	
CCU	9.88%	10.64%	
CSRU	4.64%	5.75%	

*SOFA* Sequential Organ Failure Assessment, *DMARD* disease-modifying antirheumatic drug, *ICU* intensive care unit, *CCU* coronary care unit, *CSRU* cardiac surgery recovery unit, *MICU* medical intensive care unit, *SICU* surgical intensive care unit

### Association between autoimmune disease presence and mortality

A total of 132 (26.61%) patients with autoimmune disease and 1971 (34.55%) patients without autoimmune disease died either within the hospital or in the 30 days after hospital discharge.

We analyzed the potential confounders (see the subsection “Statistical analysis and modeling” in the “Methods” section) by adding each variable into the model one at a time and evaluating how much the newly added variable affected the model estimate. We performed this analysis to reduce the number of variables within the model and to account only for those that had a meaningful affect (more than 10.00%) on model estimates. In our analysis, the SOFA score was the only variable that met the inclusion criterion for approach 2 (Table 3). Statistically adjusting for individual comorbidities included within the Elixhauser comorbidity index did not meaningfully affect model estimates (Additional file 3: Table S3). After including SOFA score in the final model, we reevaluated each potential confounder, accounting for SOFA score, and found that no other variable met the inclusion criterion (Additional file 4: Table S4). For approach 2, we only statistically adjusted for the SOFA score.

**Table 3:** Analysis of the impact of potential confounders of the autoimmune disease-30-day mortality association

Adjusting for confounders of the association between autoimmune disease and 30-day mortality	OR (95% CI)	*P* value	Magnitude of confounding
No confounders adjusted (“crude”)	0.71 (0.58–0.86)	< 0.001	–
Age adjusted	0.74 (0.60–0.90)	0.003	− 4.46%
Sex adjusted	0.71 (0.60–0.86)	< 0.001	− 0.31%
Race adjusted	0.71 (0.58–0.87)	< 0.001	− 0.93%
Documented bacteremia adjusted	0.68 (0.56–0.83)	< 0.001	4.41%
Infection site adjusted	0.72 (0.56–0.83)	0.001	− 1.39%
SOFA score adjusted	0.81 (0.65–1.00)	0.051	− 12.34%
Elixhauser comorbidity index adjusted	0.71 (0.57–0.86)	< 0.001	0.26%
DMARD or prednisone usage adjusted	0.66 (0.54–0.81)	< 0.001	7.58%
ICU care unit adjusted	0.71 (0.58–0.86)	< 0.001	0.10%

Magnitude of confounding = (OR_crude_ − OR_adjusted_)/OR_adjusted_

*OR* odds ratio, *CI* confidence interval, *SOFA* Sequential Organ Failure Assessment, *DMARD* disease-modifying antirheumatic drug, *ICU* intensive care unit

**Fig. 1:**
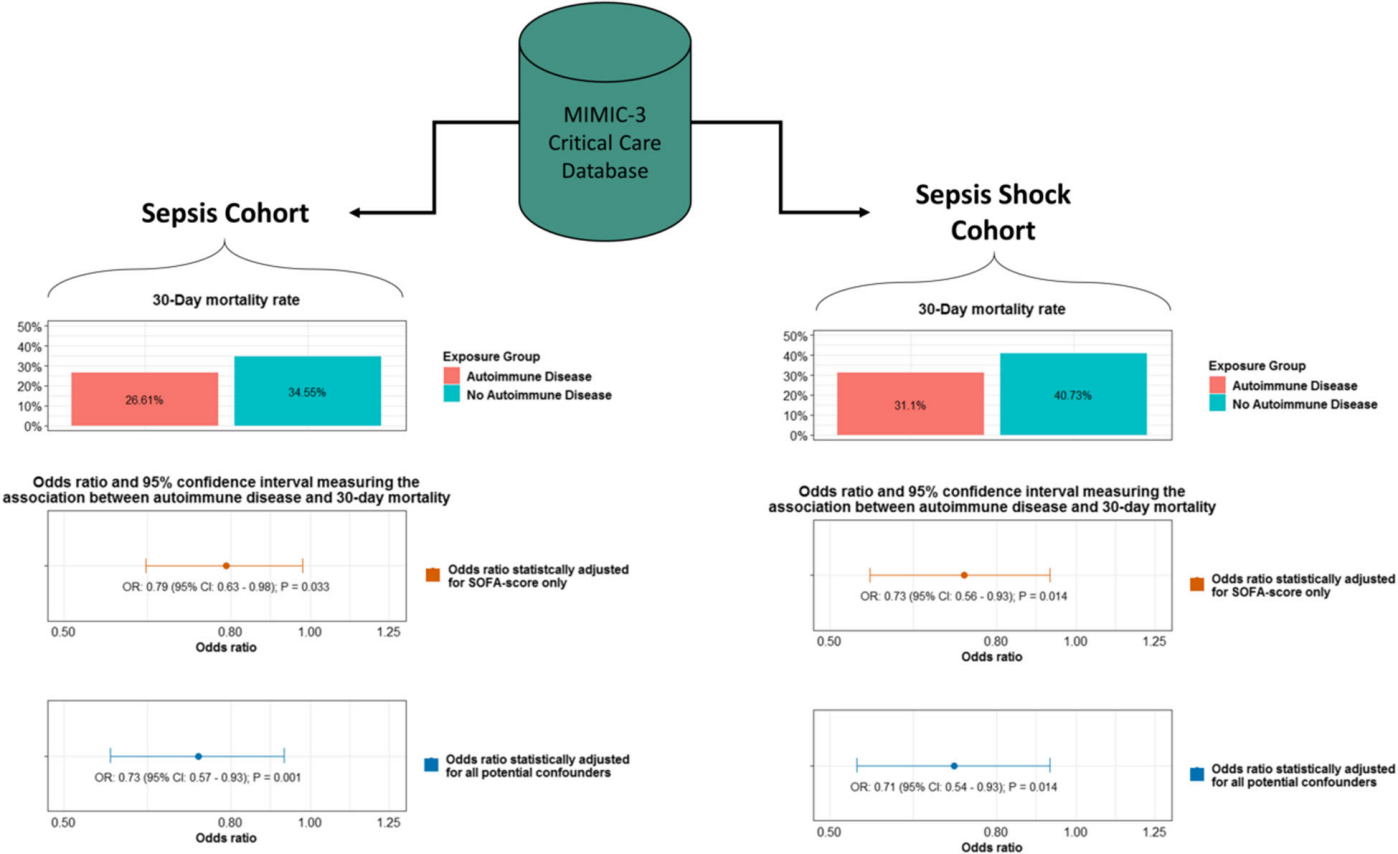
Association between autoimmune disease presence and 30-day mortality among sepsis and septic shock patients

To understand how autoimmune disease presence may influence 30-day mortality, we measured the association between autoimmune disease and 30-day mortality within the sepsis and septic shock cohorts using multivariable logistic regression models (Fig. 1 and Table 4). For the sepsis cohort, controlling for all potential confounders, autoimmune disease was associated with a statistically significant 27.00% decrease in the 30-day mortality risk (OR 0.73, 95% CI 0.57–0.93, *P* = 0.001). When only SOFA score was adjusted for, we observed a similar statistically significant reduction in the 30-day mortality risk (OR 0.79, 95% CI 0.63–0.98, *P* = 0.033).

**Table 4:** Association between autoimmune disease and 30-day mortality according to confounder adjustment strategy

Association between autoimmune disease and 30-day mortality	Sepsis cohort		Septic shock cohort	
	OR (95% CI)	*P* value	OR (95% CI)	*P* value
All potential confounders adjusted^a^	0.73 (0.57–0.93)	0.001	0.71 (0.54–0.93)	0.014
SOFA score adjusted	0.79 (0.63–0.98)	0.033	0.73 (0.56–0.93)	0.014

Sepsis cohort—ICU patients with sepsis as defined by Martin criteria. Septic shock cohort—ICU patients with sepsis as defined by Martin criteria and three consecutive mean arterial blood pressure readings below 65 mmHg in a 30-min period or at least one dose of vasopressors during the ICU stay

*OR* odds ratio, *CI* confidence interval, *SOFA* Sequential Organ Failure Assessment

^a^OR adjusted for age, sex, race, SOFA score at ICU admission, Elixhauser comorbidity index, pre-admission chronic DMARD or prednisone use, ICU care unit, documented bacteremia, and infection site

For the septic shock cohort, we observed a statistically significant 29.00% (OR 0.71, 95% CI 0.54–0.93, *P* = 0.014) and 27.00% (OR 0.73, 95% CI 0.56–0.93, P = 0.014) reduction in the 30-day mortality risk for subsequent confounder adjustment strategies.

We performed a survival analysis, using multivariable Cox Proportional-Hazards models, to assess the rate of mortality for the five most frequently reported autoimmune diseases relative to the reference group (those without autoimmune disease). For the sepsis cohort, after statistically adjusting for all potential confounders, we observed a 10.00–55.00% reduction in the 30-day mortality rate (Additional file 5: Table S5). Multiple sclerosis was the only disease where the measured association was statistically significant (HR: 0.45, 95% CI: 0.22–0.89, *p* = 0.023). For the septic shock cohort, adjusting for all potential confounders, we observed that the five most frequently reported autoimmune diseases were associated with a 10.00–66.00% reduction in the 30-day mortality rate. Once again, only multiple sclerosis was associated with a statistically significant reduction (HR 0.34, 95% CI 0.14–0.82, *p* = 0.016).

### Joint effect of autoimmune disease and chronic DMARD or prednisone use

We evaluated how immunosuppressant medications (DMARDs and prednisone) may modify the association between autoimmune disease and mortality. To do so, we measured the joint effect of autoimmune disease and chronic immunosuppressant use on 30-day mortality. In the sepsis cohort, we observed a non-statistically significant 11.00% reduction in 30-day mortality (OR 0.89, 95% CI 0.65–1.20, *P* = 0.452) (Table 4) associated with autoimmune disease patients undergoing chronic immunosuppressant therapy. A slightly stronger, yet non-statistically significant, reduction in mortality risk was observed for patients with autoimmune disease who were not on chronic immunosuppressant therapy (OR 0.82, 95% CI 0.58–1.16, *P* = 0.272). Within the septic shock cohort, we observed a similar non-statistically significant reduction in mortality risk for autoimmune disease patients undergoing chronic immunosuppressant therapy. The protective association was strongest for autoimmune disease patients not on chronic immunosuppressant therapy; however, this association was not statistically significant (OR 0.76, 95% CI 0.52–0.96, *P* = 0.029). Adjusting for SOFA score alone led to similar conclusions about the joint effect of autoimmune disease and chronic immunosuppressant use.

### Effect of autoimmune disease on 30-day mortality mediated through chronic DMARD or prednisone usage

We performed a mediation analysis where we estimated the direct and indirect effects of autoimmune disease on the 30-day mortality risk. In doing so, the mediation analysis allows us to clarify the nature of the relationship between autoimmune disease and chronic use of immunosuppressant medications to understand how autoimmune diseases are associated with mortality risk. Among sepsis patients, we observed statistically significant direct (OR 0.78, 95% CI 0.61–0.98, *P* = 0.004) and indirect (OR 1.07, 95% CI 1.01–1.13, *P* = 0.020) effects after adjusting for all confounders (Table 6). Similar direct (OR 0.75, 95% CI 0.57–0.98, *P* = 0.037) and indirect (OR 1.06, 95% CI 0.99–1.13, *P* = 0.112) effects were measured among patients in the septic shock cohort. Adjusting for SOFA score alone led to similar conclusions regarding the direct and indirect effects.

### Effect of the cytokine dysregulation on 30-day mortality

We explored additional explanations for the protective effect associated with autoimmune disease by evaluating the autoimmune disease-mortality relationship within the context of over- and underexpression of sepsis-related cytokines (Table 7). Among sepsis cohort patients and adjusting for all potential confounders, the overexpression of pro-inflammatory cytokines interleukin-12 (IL-12) and interferon-γ (INF-γ) was associated with a statistically significant 24.00% and 37.00% reduction in the 30-day mortality risk (IL-12 OR 0.76, 95% CI 0.59–0.99, *P* = 0.040; INF-γ OR 0.63, 95% CI 0.45–0.89, *P* = 0.008). We did not observe any statistically significant reduction in the 30-day mortality risk associated with the over- or underexpression of the evaluated anti-inflammatory cytokines.

Among septic shock patients, the overexpression of IL-6, IL-12, INF-γ, and TNF-α was associated with a statistically significant 23.00–41.00% reduction in 30-day mortality, adjusting for all confounders (IL-6 OR 0.75, 95% CI 0.60–0.95, *P* = 0.015; IL-12 OR 0.73, 95% CI 0.55–0.98, *P* = 0.0351; INF-γ OR 0.59, 95% CI 0.41–0.86, *P* = 0.007; TNF-α OR 0.77, 95% CI 0.61–0.96, *P* = 0.020). Additionally, the underexpression of anti-inflammatory cytokines IL-4 and IL-10 was both associated with a statistically significant reduction in the 30-day mortality risk (OR 0.62, 95% CI 0.44–0.87, *P* = 0.006).

## Discussion

This study examined the sepsis survival among ICU patients with and without autoimmune diseases using multiple analytic approaches. We used Martin criteria to identify a sepsis population in the MIMIC III database and ICD-9-CM diagnosis codes and text analysis of the patient discharge summaries to identify a subset of patients with autoimmune diseases (Table 1). Among all sepsis patients, autoimmune disease presence was associated with a statistically significant 27.00% reduction in the 30-day mortality risk (OR 0.73, 95% CI 0.57–0.93, *P* = 0.001) (Fig. 1 and Table 4). The autoimmune disease-mortality association was similar when we restricted the study population to those with septic shock (OR 0.71, 95% CI 0.54–0.93, *P* = 0.014).

In a similar study using National Inpatient Sample data, a statistically significant decrease in mortality risk was associated with Crohn’s disease (OR 0.78, 95% CI 0.63–0.97) and a small non-statistically significant decrease in risk was associated with rheumatoid arthritis (OR 0.91, 95% CI 0.82–1.01) [13]. The same study also identified a statistically significant increase in mortality risk associated with ulcerative colitis (OR 1.61, 95% CI 1.35–1.93) [13]. Our study observed a similar, but non-statistically significant reduction in the 30-day mortality risk associated with Crohn’s disease (OR 0.65, 95% CI 0.40–1.05, *P* = 0.087) and rheumatoid arthritis (OR 0.79, 95% CI 0.52–1.17, *P* = 0.247) (Additional file 6: Table S6). In contrast, our study identified a non-statistically significant reduction in the 30-day mortality risk associated with ulcerative colitis (OR 0.87, 95% CI 0.52–1.43, *P* = 0.594). The lack of statistical significance could be due to the smaller number of patients for each individual condition.

A secondary but important result of this study addresses how medications associated with autoimmune disease treatment may impact the observed association. We observed that patients with autoimmune disease and on immunosuppressant therapy had similar reductions in the 30-day mortality risk (OR 0.89, 95% CI 0.65–1.20, *P* = 0.452) as autoimmune patients not on immunosuppressant therapy (OR 0.82, 95% CI 0.58–1.16, *P* = 0.272) (Table 5). This finding indicates that these medications did not modify the association between autoimmune disease and 30-day mortality. The results of the mediation analysis (Table 6) provided further evidence of the limited impact of these medications on the autoimmune disease-mortality relationship (indirect effect OR 1.07, 95% CI 1.01–1.13, *P* = 0.020). Altogether, these findings suggest that autoimmune diseases affect 30-day mortality through a mechanism unrelated to chronic DMARD or prednisone use.

**Table 5:** Joint effect of autoimmune disease and DMARD or prednisone use on 30-day mortality

Association between autoimmune disease and 30-day mortality, accounting for chronic DMARD and prednisone use	Sepsis cohort		Septic shock cohort	
	OR (95% CI)	*P* value	OR (95% CI)	*P* value
All potential confounders adjusted^a^				
No autoimmune disease presence				
No chronic DMARD/prednisone usage	1.00	Reference	1.00	Reference
Autoimmune disease presence				
No chronic DMARD or prednisone usage	0.82 (0.58–1.16)	0.272	0.76 (0.50–1.14)	0.186
Chronic DMARD or prednisone usage	0.89 (0.65–1.20)	0.452	0.87 (0.62–1.22)	0.428
SOFA score adjusted				
No autoimmune disease presence				
No chronic DMARD/prednisone usage	1.00	Reference	1.00	Reference
Autoimmune disease presence				
No chronic DMARD or prednisone usage	0.80 (0.57–1.11)	0.190	0.73 (0.49–1.07)	0.114
Chronic DMARD or prednisone usage	0.84 (0.62–1.12)	0.231	0.77 (0.55–1.07)	0.121

Sepsis cohort—ICU patients with sepsis as defined by Martin criteria. Septic shock cohort—ICU patients with sepsis as defined by Martin criteria and three consecutive mean arterial blood pressure readings below 65 mmHg in a 30-min period or at least one dose of vasopressors during the ICU stay

*OR* odds ratio, *CI* confidence interval, *DMARD* disease-modifying antirheumatic drug, *SOFA* Sequential Organ Failure Assessment

^a^OR adjusted for age, sex, race, SOFA score at ICU admission, Elixhauser comorbidity index, pre-admission chronic DMARD or prednisone use, ICU care unit, documented bacteremia, and infection site

**Table 6:** Direct and Indirect effects of autoimmune disease on 30-day mortality, mediated through immunomodulation medication use

Mediation analysis of the effect of autoimmune disease on 30-day mortality	Sepsis cohort		Septic shock cohort	
	OR (95% CI)	P-value	OR (95% CI)	*P* value
All potential confounders adjusted^a^				
Indirect effect	1.07 (1.01–1.13)	0.020	1.06 (0.99–1.13)	0.112
Direct effect	0.78 (0.61–0.98)	0.004	0.75 (0.57–0.98)	0.037
SOFA adjusted				
Indirect effect	1.06 (1.00–1.11)	0.033	1.04 (0.99–1.12)	0.130
Direct effect	0.75 (0.60–0.93)	0.011	0.69 (0.53–0.90)	0.006

Sepsis cohort—ICU patients with sepsis defined with Martin criteria. Septic shock cohort—ICU patients with sepsis defined by Martin criteria and three consecutive mean arterial blood pressure readings below 65 mmHg in a 30-min period or at least one dose of vasopressors during the ICU stay

*OR* odds ratio, *CI* confidence interval, *DMARD* disease-modifying antirheumatic drug

^a^OR adjusted for age, sex, race, SOFA score at ICU admission, Elixhauser comorbidity index, pre-admission chronic DMARD or prednisone use, ICU care unit, documented bacteremia, and infection site

We evaluated the possible effect of cytokine dysregulation on 30-day mortality to understand other potential mechanisms that may explain the observed associations (Table 7). Autoimmune diseases associated with the overexpression of pro-inflammatory cytokines IL-12 and INF-γ were associated with a statistically significant reduction in the 30-day mortality risk (IL-12 OR 0.76, 95% CI 0.59–0.99, *P* = 0.040; INF-γ OR 0.63, 95% CI 0.45–0.89, *P* = 0.008). These results highlight how the immunosuppressive state induced by sepsis may play a critical role in mortality among sepsis patients [3] and that therapies that augment IL-12 and INF-γ expression may improve sepsis survival [32].

**Table 7:** Association between the dysregulation of cytokines and specific autoimmune diseases

Pathway	Cytokine dysregulation (based upon literature)		Diseases involved	Sepsis cohort				Septic shock cohort			
				Model 1		Model 2		Model 1		Model 2	
				OR (95% CI)	*P* value	OR (95% CI)	*P* value	OR (95% CI)	*P* value	OR (95% CI)	*P* value
Pro-inflammatory	Overexpression	IL-1 [8, 9, 12]	Crohn’s disease, ulcerative colitis, inflammatory myopathies*, giant cell arteritis	0.81 (0.62–1.06)	0.126	0.75 (0.57–0.97)	0.032	0.80 (0.59–1.07)	0.135	0.75 (0.56–1.01)	0.062
		IL-6 [3, 8, 9, 12]	Rheumatoid arthritis, Crohn’s disease, ulcerative colitis, multiple sclerosis, systemic sclerosis, giant cell arteritis	0.83 (0.68–1.01)	0.066	0.85 (0.70–1.04)	0.116	0.75 (0.60–0.95)	0.015	0.77 (0.62–0.97)	0.025
		IL-12 [8, 12]	Crohn’s disease, ulcerative colitis, multiple sclerosis	0.76 (0.59–0.99)	0.040	0.71 (0.55–0.92)	0.009	0.73 (0.55–0.98)	0.035	0.69 (0.52–0.92)	0.012
		INF-y [8, 9, 12]	Crohn’s disease, ulcerative colitis, multiple sclerosis, giant cell arteritis	0.63 (0.45–0.89)	0.008	0.59 (0.42–0.83)	0.002	0.59 (0.41–0.86)	0.007	0.56 (0.39–0.82)	0.003
		TNF-a [3, 8, 9, 12]	Crohn’s disease, ulcerative colitis, rheumatoid arthritis, psoriatic arthritis, multiple sclerosis, systemic sclerosis, scleroderma, inflammatory myopathies*, giant cell arteritis	0.84 (0.69–1.02)	0.077	0.86 (0.71–1.04)	0.120	0.77 (0.61–0.96)	0.020	0.79 (0.63–0.98)	0.032
	Underexpression	IL-12 [8, 11]	Myasthenia gravis	0.31 (0.08–1.22)	0.094	0.37 (0.09–1.48)	0.159	0.24 (0.03–1.67)	0.148	0.27 (0.04–1.91)	0.189
		INF-y [8, 12]	Systemic lupus erythematosus	0.88 (0.52–1.51)	0.645	0.75 (0.45–1.28)	0.294	0.90 (0.53–1.55)	0.708	0.79 (0.47–1.34)	0.389
		TNF-a [8, 9]	Systemic lupus erythematosus and ankylosing spondylitis	0.83 (0.52–1.33)	0.443	0.71 (0.44–1.13)	0.145	0.86 (0.54–1.39)	0.549	0.75 (0.47–1.20)	0.236
Anti-inflammatory	Overexpression	IL-4 [3, 8, 12]	Systemic lupus erythematosus, systemic sclerosis, scleroderma	1.02 (0.64–1.62)	0.941	0.88 (0.56–1.38)	0.571	1.03 (0.64–1.66)	0.908	0.92 (0.58–1.46)	0.712
		IL-10 [9, 12]	Crohn’s disease, ulcerative colitis, systemic lupus e	0.85 (0.66–1.10)	0.220	0.77 (0.60–0.99)	0.039	0.85 (0.65–1.11)	0.239	0.79 (0.61–1.03)	0.083
		TGF-B [12]	Inflammatory myopathies*	0.62 (0.15–2.49)	0.500	0.53 (0.13–2.13)	0.371	0.83 (0.21–3.37)	0.799	0.80 (0.20–3.21)	0.754
		IL-13 [3, 8]	Multiple sclerosis, systemic sclerosis, scleroderma	0.63 (0.37–1.06)	0.084	0.62 (0.36–1.04)	0.072	0.55 (0.29–1.02)	0.058	0.53 (0.28–0.98)	0.043
	Underexpression	IL-1 receptor antagonist [9]	Rheumatoid arthritis and psoriatic arthritis	0.90 (0.67–1.21)	0.492	1.11 (0.83–1.47)	0.481	0.76 (0.53–1.07)	0.118	0.90 (0.64–1.27)	0.555
		IL-4 [12]	Rheumatoid arthritis and multiple sclerosis	0.76 (0.57–1.00)	0.054	0.89 (0.67–1.18)	0.411	0.62 (0.44–0.87)	0.006	0.70 (0.50–0.98)	0.040
		IL-10 [8, 12]	Rheumatoid arthritis and multiple sclerosis	0.76 (0.57–1.00)	0.054	0.89 (0.67–1.18)	0.411	0.62 (0.44–0.87)	0.006	0.70 (0.50–0.98)	0.040
		TGF-B [8, 12]	Systemic lupus erythematosus	0.88 (0.52–1.51)	0.645	0.75 (0.45–1.28)	0.294	0.90 (0.53–1.55)	0.708	0.79 (0.47–1.34)	0.389

Model 1 adjusted for age, sex, race, ICU unit, Elixhauser score, pre-admission chronic DMARD or prednisone use, and SOFA. Model 2 adjusted for SOFA

*Inflammatory myopathies include polymyositis, dermatomyositis, and inclusion body myositis

The observed relationships between cytokine dysregulation and mortality are further supported by previous studies identifying that the sepsis response impairs INF-y production [33], monocyte expression of HLA-DR and production of leukocyte cytokines (e.g., IL-1, IL-6, and TNF-a) [34, 35], and macrophage phagocytic and microbicidal activity and their ability to produce pro-inflammatory cytokines IL-12 and TNF-a [36]. This sepsis-induced immunosuppressive state may be further mediated by the release of anti-inflammatory cytokines (e.g., IL-4 and IL-10) [33]. IL-4 has been shown to reduce pro-inflammatory cytokine expression and increase the expression of other anti-inflammatory cytokines [37, 38] while IL-10 has been shown to downregulate the expression of several pro-inflammatory cytokines and impair HLA-DR expression [33, 39]. Patients who experience pre-sepsis over- or underexpression of specific cytokines may be better suited to survive the immune function impairment [33, 40–44]. The high rates of nosocomial infections (especially of the lung) and mortality among sepsis patients [33, 45] further highlight the clinical importance of the immune function impairment.

There are several limitations to our study. First, our results may be affected by diagnostic bias, where patients with autoimmune disease are admitted to the ICU earlier than the rest of the population due to the severity of the autoimmune diagnosis and the immunocompromised status, thus potentially resulting in better survival. This selection process would result in higher prevalence of patients with autoimmune disease among ICU population. However, the autoimmune disease prevalence among patients in our study was 8.00% which is at the higher end of the estimated prevalence of autoimmune diseases in the general population, estimated between 3.00 and 10.00% [46–49]. The observed prevalence can be explained by the significantly higher than average age of the ICU population (mean age is 66.07 years) since autoimmune disease prevalence increases with increasing age [50]. In our analysis, we observed that patients with autoimmune disease had a significantly lower SOFA score than patients without autoimmune disease implying that autoimmune disease patients enter the ICU in better condition (5.63 ± 3.67 vs. 6.50 ± 3.80, *P* < 0.001). To account for this difference, we statistically adjusted for SOFA score in all analyses, thus eliminating this bias. To further explore the potential role of diagnostic bias, we estimated the relationship between the presence of other comorbidities involving immunosuppression factors (AIDS, lymphoma, metastatic cancer, and solid-state tumors) and 30-day mortality (Additional file 7: Table S7). We found that for these patients, the 30-day mortality risk was higher (OR 1.98, 95% CI 1.65–2.36, *P* ≤ 0.001). We believe that further studies with more diverse pre-ICU admission data will help to fully rule out diagnostic bias.

Another limitation of the study stems from the fact that MIMIC database is based on a single-center study institution, and the findings may not be generalizable. The confidence in these results comes from the initial analysis of MIMIC II data, covering half of the time of MIMIC III data. Analysis on half of the population currently available led to similar conclusions. Lastly, we may not have accounted for some important confounding factors, though every attempt was made to include them in the study. Specifically, it is hard to account for past medical history with high certainty. To overcome this limitation, we performed an automated and manual analysis of discharge summaries. Manual evaluation of a subset of discharge summaries revealed some levels of ambiguity or missed information about patient’s condition as compared to automated analysis that requires further exploration. We could not measure the level of uncertainty due to the large number of cases in the study, referring such analysis to further studies measuring the uncertainly related to information extraction from free text.

## Conclusion

We demonstrated that select autoimmune diseases are associated with a lower 30-day mortality risk among sepsis patients in the ICU. This association was nearly identical among patients with a more severe form of sepsis. The primary driver of mortality among sepsis patients is still an open question [51]. Our study found that autoimmune disease-induced dysregulation of sepsis-related cytokines (specifically IL-12 and INF-γ) prior to sepsis onset was associated with a lower 30-day mortality risk. These results provide further support regarding the potential benefit of therapies designed to augment the production of IL-12, INF-γ, and other pro-inflammatory cytokines in the treatment of sepsis [32]. Further research utilizing data from other medical centers will provide further validation and insight into these findings and potential causal mechanisms.

## Additional files

Additional file 1: **Table S1.** Dysregulated cytokines and specific autoimmune diseases included in the study’s autoimmune definition. (DOCX 16 kb)

Additional file 2: **Table S2.** Biologic and conventional DMARD medications included in the present study. (DOCX 13 kb)

Additional file 3: **Table S3.** Analysis of the impact of individual comorbidities on the autoimmune disease-30-day mortality association. (DOCX 15 kb)

Additional file 4: **Table S4.** Impact of adjusting for potential confounders and SOFA score on the autoimmune disease-30-day mortality association. (DOCX 15 kb)

Additional file 5: **Table S5.** Survival analysis of the five most frequently reported autoimmune diseases. (DOCX 13 kb)

Additional file 6: **Table S6.** Association between each autoimmune disease and 30-day mortality. (DOCX 14 kb)

Additional file 7: **Table S7.** Sensitivity analysis exploring the potential effect of diagnostic bias among other immunocompromised patients. (DOCX 12 kb)

## Abbreviations

CI: Confidence interval
DMARD: Disease-modifying antirheumatic drug
HR: Hazards ratio
ICU: Intensive care unit
IL: Interleukin
INF-γ: Interferon gamma
MIMIC: Multiparameter Intelligent Monitoring in Intensive Care
OR: Odds ratio
SOFA: Sequential Organ Failure Assessment
TNF-α: Tumor necrosis factor alpha
