# Supplementary material for: The association between autoimmune disease and 30-day mortality among sepsis ICU patients: a cohort study
Source: Crit Care. 2019 Mar 18;23:93. doi: 10.1186/s13054-019-2357-1 (PMC6423870; doi:10.1186/s13054-019-2357-1)
Supplement: Supplementary file 1 — Table S1. Dysregulated cytokines and specific autoimmune diseases included in the study’s autoimmune definition. (DOCX 16 kb) [file 13054_2019_2357_MOESM1_ESM.docx]

**Table S1: Dysregulated cytokines and specific autoimmune diseases included in the study’s autoimmune definition**

| **Autoimmune Disease** | **Overexpressed Cytokines** | | **Underexpressed Cytokines** | |
| --- | --- | --- | --- | --- |
|  | **Pro-inflammatory** | **Anti-inflammatory** | **Pro-inflammatory** | **Anti-inflammatory** |
| Rheumatoid arthritis | IL-6 and TNF-α [3,8,9] |  |  | IL-1 Receptor Antagonist, IL-4, and IL-10 [9,12] |
| Crohn’s disease | IL-1, IL-6, IL-12, INF-γ,  and TNF-α [3,8,9] | IL-10 [9] |  |  |
| Ulcerative colitis | IL-1, IL-6, IL-12, INF-γ,  and TNF-α [3,8,9] | IL-10 [9] |  |  |
| Multiple sclerosis | IL-6, IL-12, INF-γ,  TNF-α [8,12] | IL-13 [8] |  | IL-4 and IL-10 [8,12] |
| Systemic lupus erythematosus |  | IL-4 and IL-10 [12] | INF-γ and TNF-α [8,12] | TGF-β [8,12] |
| Ankylosing spondylitis |  |  | TNF-α [8] |  |
| Psoriatic arthritis | TNF-α [3,8,9] |  |  | IL-1 Receptor Antagonist [9] |
| Myasthenia gravis |  |  | IL-12 [8,11] |  |
| Giant Cell Arteritis | IL-6, INF-y,  and TNF-α [9] |  |  |  |
| Scleroderma | TNF-α [8] | IL-4 and IL-10 [12] |  |  |
| Systemic Sclerosis | IL-6 and TNF-α [8] | IL-4 and IL-13 [8] |  |  |
| Polymyositis | IL-1 and TNF-α [12] | TGF-β [12] |  |  |
| Dermatomyositis | IL-1 and TNF-α [12] | TGF-β [12] |  |  |
| Inclusion Body Myositis | IL-1 and TNF-α [12] | TGF-β [12] |  |  |

Abbreviations: IL, Interleukin; TNF, Tumor Necrosis Factor; INF, Interferon
