# Supplementary material for: The association between autoimmune disease and 30-day mortality among sepsis ICU patients: a cohort study
Source: Crit Care. 2019 Mar 18;23:93. doi: 10.1186/s13054-019-2357-1 (PMC6423870; doi:10.1186/s13054-019-2357-1)
Supplement: Supplementary file 2 — Table S2. Biologic and conventional DMARD medications included in the present study. (DOCX 13 kb) [file 13054_2019_2357_MOESM2_ESM.docx]

**Table S2: Biologic and conventional DMARD medications included in the present study**

| **Medication** | **Biologic DMARD** |
| --- | --- |
| Abatacept | Yes |
| Adalimumab | Yes |
| Anakinra | Yes |
| Etanercept | Yes |
| Golimumab | Yes |
| Infliximab | Yes |
| Rituximab | Yes |
| Tocilizumab | Yes |
| Azathioprine | No |
| Chloroquine | No |
| Cyclosporine | No |
| Penicillamine | No |
| Auranofin | No |
| Hydroxychloroquine | No |
| Leflunomide | No |
| Methotrexate | No |
| Minocycline | No |
| Sulfasalazine | No |
| Tofacitinib | No |
| Cyclophosphamide | No |

Medications listed in table are for the non-proprietary name only.
