# Supplementary material for: The association between autoimmune disease and 30-day mortality among sepsis ICU patients: a cohort study
Source: Crit Care. 2019 Mar 18;23:93. doi: 10.1186/s13054-019-2357-1 (PMC6423870; doi:10.1186/s13054-019-2357-1)
Supplement: Supplementary file 3 — Table S3. Analysis of the impact of individual comorbidities on the autoimmune disease-30-day mortality association. (DOCX 15 kb) [file 13054_2019_2357_MOESM3_ESM.docx]

**Table S3: Analysis of the impact of individual comorbidities on the autoimmune disease-30-day mortality association.**

| **Model adjusted for individual comorbidities from the Elixhauser score** | **OR (95% CI)** | **p-value** | **Magnitude of Confounding** |
| --- | --- | --- | --- |
|  |  |  |  |
| No confounders adjusted ("crude") | 0.71 (0.58 - 0.86) | <0.001 | -- |
| Congestive Heart Failure | 0.72 (0.59 - 0.87) | 0.001 | -1.39% |
| Rheumatoid Arthritis/Collagen | 0.70 (0.55 - 0.87) | 0.002 | 1.43% |
| Other Neurological Disorders | 0.72 (0.59 - 0.87) | 0.001 | -1.39% |
| Alcohol Abuse | 0.70 (0.58 - 0.86) | <0.001 | 1.43% |
| Diabetes with Complications | 0.70 (0.57 - 0.85) | <0.001 | 1.43% |
| Weight loss | 0.70 (0.57 - 0.85) | <0.001 | 1.43% |
| Deficiency Anemia | 0.72 (0.58 - 0.87) | 0.001 | -1.39% |
| Diabetes uncomplicated | 0.71 (0.58 - 0.86) | <0.001 | 0.00% |
| Pulmonary Circulation Disorders | 0.70 (0.57 - 0.85) | <0.001 | 1.43% |
| Peptic Ulcer Disease Excluding Bleeding | 0.71 (0.58 - 0.86) | <0.001 | 0.00% |
| Depression | 0.71 (0.58 - 0.87) | <0.001 | 0.00% |
| Cardiac arrhythmia | 0.71 (0.58 - 0.87) | 0.001 | 0.00% |
| Metastatic cancer | 0.72 (0.59 - 0.87) | 0.001 | -1.39% |
| Peripheral vascular disorders | 0.71 (0.58 - 0.86) | <0.001 | 0.00% |
| Valvular Disease | 0.71 (0.58 - 0.86) | <0.001 | 0.00% |
| Drug abuse | 0.70 (0.57 - 0.85) | <0.001 | 1.43% |
| Coagulopathy | 0.71 (0.58 - 0.87) | <0.001 | 0.00% |
| Obesity | 0.71 (0.58 - 0.86) | <0.001 | 0.00% |
| Blood Loss Anemia | 0.71 (0.58 - 0.86) | <0.001 | 0.00% |
| Liver disease | 0.71 (0.58 - 0.86) | <0.001 | 0.00% |
| Hypothyroidism | 0.71 (0.58 - 0.86) | <0.001 | 0.00% |
| Psychoses | 0.71 (0.58 - 0.86) | <0.001 | 0.00% |
| Chronic pulmonary disease | 0.71 (0.58 - 0.86) | <0.001 | 0.00% |
| Renal failure | 0.71 (0.58 - 0.86) | <0.001 | 0.00% |
| Solid tumor without metastasis | 0.71 (0.58 - 0.86) | <0.001 | 0.00% |
| Fluid and Electrolyte Disorders | 0.71 (0.58 - 0.86) | <0.001 | 0.00% |
| HIV/AIDS | 0.71 (0.58 - 0.86) | <0.001 | 0.00% |
| Hypertension | 0.71 (0.58 - 0.86) | <0.001 | 0.00% |
| Paralysis | 0.71 (0.58 - 0.86) | <0.001 | 0.00% |
| Lymphoma | 0.71 (0.58 - 0.86) | <0.001 | 0.00% |
