# Supplementary material for: The association between autoimmune disease and 30-day mortality among sepsis ICU patients: a cohort study
Source: Crit Care. 2019 Mar 18;23:93. doi: 10.1186/s13054-019-2357-1 (PMC6423870; doi:10.1186/s13054-019-2357-1)
Supplement: Supplementary file 4 — Table S4. Impact of adjusting for potential confounders and SOFA score on the autoimmune disease-30-day mortality association. (DOCX 15 kb) [file 13054_2019_2357_MOESM4_ESM.docx]

**Table S4: Impact of adjusting for potential confounders and SOFA score on the autoimmune disease-30-day mortality association.**

| **Adjusted For** | **OR (95% CI)** | **p-value** | **Magnitude of Confounding** |
| --- | --- | --- | --- |
| sofa | 0.81 (0.65 - 1.00) | 0.051 | -- |
| sofa + Age | 0.86 (0.69 - 1.06) | 0.161 | -5.48% |
| sofa + Sex | 0.80 (0.65 - 0.99) | 0.044 | 0.78% |
| sofa + Race | 0.81 (0.65 - 1.00) | 0.054 | -0.11% |
| sofa + ICU care unit | 0.81 (0.65 - 1.00) | 0.051 | 0.01% |
| sofa + Elixhauser | 0.80 (0.65 - 0.99) | 0.045 | 0.90% |
| sofa + Congestive Heart Failure | 0.82 (0.66 - 1.01) | 0.069 | -1.39% |
| sofa + Cardiac arrhythmia | 0.82 (0.66 - 1.01) | 0.067 | -1.18% |
| sofa + Valvular Disease | 0.81 (0.66 - 1.00) | 0.055 | -0.30% |
| sofa + Pulmonary Circulation Disorders | 0.81 (0.65 - 0.99) | 0.044 | 0.67% |
| sofa + Peripheral vascular disorders | 0.82 (0.66 - 1.01) | 0.060 | -0.78% |
| sofa + Hypertension | 0.81 (0.65 - 1.00) | 0.050 | 0.14% |
| sofa + Paralysis | 0.81 (0.65 - 1.00) | 0.051 | 0.04% |
| sofa + Other Neurological Disorders | 0.82 (0.66 - 1.01) | 0.061 | -0.81% |
| sofa + Chronic pulmonary disease | 0.81 (0.66 - 1.00) | 0.053 | -0.17% |
| sofa + Diabetes uncomplicated | 0.81 (0.66 - 1.00) | 0.051 | -0.04% |
| sofa + Diabetes with Complications | 0.81 (0.65 - 0.99) | 0.044 | 0.65% |
| sofa + Hypothyroidism | 0.81 (0.65 - 1.00) | 0.048 | 0.29% |
| sofa + Renal failure | 0.81 (0.65 - 1.00) | 0.049 | 0.20% |
| sofa + Liver disease | 0.81 (0.66 - 1.00) | 0.052 | -0.06% |
| sofa + Peptic Ulcer Disease Excluding Bleeding | 0.81 (0.65 - 1.00) | 0.047 | 0.35% |
| sofa + HIV/AIDS | 0.81 (0.66 - 1.00) | 0.051 | -0.04% |
| sofa + Lymphoma | 0.81 (0.65 - 1.00) | 0.051 | 0.01% |
| sofa + Metastatic cancer | 0.83 (0.67 - 1.03) | 0.088 | -2.43% |
| sofa + Solid tumor without metastasis | 0.81 (0.65 - 1.00) | 0.051 | 0.00% |
| sofa + Rheumatoid Arthritis/Collagen | 0.77 (0.61 - 0.99) | 0.040 | 4.66% |
| sofa + Coagulopathy | 0.81 (0.65 - 1.00) | 0.049 | 0.15% |
| sofa + Obesity | 0.81 (0.66 - 1.00) | 0.057 | -0.51% |
| sofa + Weight loss | 0.80 (0.65 - 0.99) | 0.043 | 0.84% |
| sofa + Fluid and Electrolyte Disorders | 0.81 (0.65 - 1.00) | 0.050 | 0.07% |
| sofa + Blood Loss Anemia | 0.81 (0.66 - 1.00) | 0.053 | -0.17% |
| sofa + Deficiency Anemia | 0.82 (0.66 - 1.01) | 0.067 | -1.26% |
| sofa + Alcohol Abuse | 0.80 (0.65 - 0.99) | 0.039 | 1.31% |
| sofa + Drug abuse | 0.80 (0.65 - 0.99) | 0.041 | 1.05% |
| sofa + Psychoses | 0.81 (0.66 - 1.00) | 0.052 | -0.08% |
| sofa + Depression | 0.81 (0.66 - 1.00) | 0.055 | -0.33% |
| sofa + SAPS | 0.80 (0.64 - 0.98) | 0.038 | 1.83% |
| sofa + APACHE-III | 0.82 (0.66 - 1.02) | 0.076 | -1.67% |
| sofa + OASIS | 0.82 (0.67 - 1.02) | 0.073 | -1.62% |
| Sofa + DMARD or Prednisone usage | 0.75 (0.61 – 0.93) | 0.011 | 8.00% |
