# Supplementary material for: The association between autoimmune disease and 30-day mortality among sepsis ICU patients: a cohort study
Source: Crit Care. 2019 Mar 18;23:93. doi: 10.1186/s13054-019-2357-1 (PMC6423870; doi:10.1186/s13054-019-2357-1)
Supplement: Supplementary file 5 — Table S5. Survival analysis of the five most frequently reported autoimmune diseases. (DOCX 13 kb) [file 13054_2019_2357_MOESM5_ESM.docx]

**Table S5: Survival analysis of the five most frequently reported autoimmune diseases.**

|  |  |  | **Model 1** | |  | **Model 2** | |
| --- | --- | --- | --- | --- | --- | --- | --- |
| **Sepsis Cohort** | **N** |  | **HR (95%CI)** | **P-value** |  | **HR (95%CI)** | **P-value** |
| Rheumatoid arthritis | 130 |  | 0.87 (0.64 - 1.19) | 0.382 |  | 1.09 (0.81 - 1.48) | 0.568 |
| Crohn’s disease | 114 |  | 0.73 (0.49 - 1.09) | 0.125 |  | 0.64 (0.43 - 0.96) | 0.032 |
| Ulcerative colitis | 86 |  | 0.90 (0.62 - 1.32) | 0.607 |  | 0.84 (0.58 - 1.23) | 0.379 |
| Multiple sclerosis | 64 |  | 0.45 (0.22 - 0.89) | 0.023 |  | 0.44 (0.22 - 0.88) | 0.020 |
| Systemic lupus erythematosus | 52 |  | 0.88 (0.52 - 1.51) | 0.645 |  | 0.75 (0.45 - 1.28) | 0.294 |
| **Septic Shock Cohort** |  |  |  |  |  |  |  |
| Rheumatoid arthritis | 92 |  | 0.72 (0.50 - 1.04) | 0.080 |  | 0.87 (0.61 - 1.25) | 0.446 |
| Crohn’s disease | 85 |  | 0.72 (0.48 - 1.10) | 0.127 |  | 0.68 (0.45 - 1.03) | 0.069 |
| Ulcerative colitis | 57 |  | 0.89 (0.57 - 1.39) | 0.610 |  | 0.82 (0.53 - 1.27) | 0.372 |
| Multiple sclerosis | 40 |  | 0.34 (0.14 - 0.82) | 0.016 |  | 0.32 (0.13 - 0.77) | 0.011 |
| Systemic lupus erythematosus | 40 |  | 0.90 (0.53 - 1.55) | 0.708 |  | 0.79 (0.47 - 1.34) | 0.389 |

Model 1 adjusted for age, sex, race, SOFA score at ICU admission, Elixhauser comorbidity index, pre-admission chronic DMARD or prednisone use, ICU care unit, documented bacteremia, infection site
Model 2 adjusted for SOFA score
