# Supplementary material for: The association between autoimmune disease and 30-day mortality among sepsis ICU patients: a cohort study
Source: Crit Care. 2019 Mar 18;23:93. doi: 10.1186/s13054-019-2357-1 (PMC6423870; doi:10.1186/s13054-019-2357-1)
Supplement: Supplementary file 6 — Table S6. Association between each autoimmune disease and 30-day mortality. (DOCX 14 kb) [file 13054_2019_2357_MOESM6_ESM.docx]

**Table S6: Association between each autoimmune disease and 30-day mortality.**

| **Autoimmune Condition** | **Sepsis Cohort** | | | | **Septic Shock Cohort** | | | |
| --- | --- | --- | --- | --- | --- | --- | --- | --- |
|  | **Model 1** | | **Model 2** | | **Model 1** | | **Model 2** | |
|  | **OR (95% CI)** | **P-value** | **OR (95% CI)** | **P-value** | **OR (95% CI)** | **P-value** | **OR (95% CI)** | **P-value** |
| Rheumatoid arthritis | 1.06 (0.72 - 1.53) | 0.772 | 0.79 (0.52 - 1.17) | 0.247 | 0.77 (0.49 - 1.19) | 0.253 | 0.61 (0.37 - 0.97) | 0.042 |
| Crohn’s disease | 0.58 (0.36 - 0.90) | 0.020 | 0.65 (0.40 - 1.05) | 0.087 | 0.55 (0.33 - 0.89) | 0.019 | 0.57 (0.33 - 0.95) | 0.036 |
| Ulcerative colitis | 0.85 (0.52 - 1.35) | 0.494 | 0.87 (0.52 - 1.43) | 0.594 | 0.72 (0.40 - 1.26) | 0.259 | 0.78 (0.42 - 1.40) | 0.406 |
| Multiple sclerosis | 0.49 (0.23 - 0.92) | 0.036 | 0.47 (0.22 - 0.90) | 0.031 | 0.38 (0.16 - 0.80) | 0.017 | 0.40 (0.17 - 0.84) | 0.023 |
| Systemic lupus erythematosus | 0.74 (0.38 - 1.39) | 0.365 | 0.90 (0.44 - 1.75) | 0.76 | 0.81 (0.40 - 1.56) | 0.533 | 0.93 (0.44 - 1.89) | 0.844 |
| Ankylosing spondylitis | 0.57 (0.16 - 1.56) | 0.320 | 0.73 (0.20 - 2.05) | 0.583 | 0.64 (0.18 - 1.88) | 0.452 | 0.81 (0.22 - 2.46) | 0.728 |
| Psoriatic arthritis | 1.07 (0.36 - 2.79) | 0.890 | 1.05 (0.33 - 2.97) | 0.936 | 1.02 (0.26 - 3.41) | 0.972 | 1.14 (0.26 - 4.26) | 0.847 |
| Myasthenia gravis | 0.32 (0.05 - 1.17) | 0.136 | 0.24 (0.04 - 0.91) | 0.067 | 0.21 (0.01 - 1.17) | 0.145 | 0.18 (0.01 - 1.03) | 0.110 |
| Giant Cell Arteritis | 0.36 (0.06 - 1.34) | 0.187 | 0.29 (0.04 - 1.10) | 0.112 | NA | NA | NA | NA |
| Scleroderma | 1.41 (0.43 - 4.39) | 0.555 | 1.45 (0.44 - 4.53) | 0.526 | 1.10 (0.27 - 4.03) | 0.892 | 1.17 (0.28 - 4.44) | 0.819 |
| Systemic Sclerosis | 2.98 (0.75 - 12.84) | 0.122 | 3.27 (0.80 - 14.88) | 0.105 | 4.27 (0.86 - 31.06) | 0.094 | 4.17 (0.80 - 31.69) | 0.111 |
| Inflammatory Myopathies* | 0.84 (0.21 - 2.71) | 0.781 | 1.00 (0.24 - 3.41) | 0.999 | 0.95 (0.18 - 4.54) | 0.948 | 0.99 (0.18 - 4.90) | 0.990 |

*Inflammatory Myopathies includes Polymyositis, Dermatomyositis, and Inclusion Body Myositis
 Model 1 adjusted for SOFA
Model 2 adjusted for: Age, Sex, Race, ICU Unit, Elixhauser score, pre-admission chronic DMARD or prednisone use, SOFA
