# Supplementary material for: The association between autoimmune disease and 30-day mortality among sepsis ICU patients: a cohort study
Source: Crit Care. 2019 Mar 18;23:93. doi: 10.1186/s13054-019-2357-1 (PMC6423870; doi:10.1186/s13054-019-2357-1)
Supplement: Supplementary file 7 — Table S7. Sensitivity analysis exploring the potential effect of diagnostic bias among other immunocompromised patients. (DOCX 12 kb) [file 13054_2019_2357_MOESM7_ESM.docx]

**Table S7: Sensitivity analysis exploring the potential effect of diagnostic bias among other immunocompromised patients.**

| **Association between other immunocompromised ^A^ patients and 30-day mortality** |  | **Sepsis Cohort** | |  | **Septic Shock Cohort** | |
| --- | --- | --- | --- | --- | --- | --- |
|  |  | **OR (95%CI)** | **P-value** |  | **OR (95%CI)** | **P-value** |
| All potential confounders adjusted ^B^ |  | 1.98 (1.65 – 2.36) | < 0.001 |  | 1.66 (1.35 – 2.05) | < 0.001 |
| SOFA score adjusted |  | 2.45 (2.09 – 2.88) | < 0.001 |  | 2.11 (1.75 – 2.56) | < 0.001 |

^A^ Immunocompromised patients (N = 532; 30-day mortality = 54.51%) were defined as patients with the following immunosuppressive conditions: HIV/AIDS (N= 13), lymphoma (N = 106), metastatic cancer (N = 247), and solid-state tumors (N = 174)
^B^ OR adjusted for: age, sex, race, SOFA score at ICU admission, Elixhauser comorbidity index, pre-admission chronic DMARD or prednisone use, ICU care unit, Documented bacteremia and infection site
